# Innovation of Human Body Positioning System and Basketball Training System

**DOI:** 10.1155/2022/2369925

**Published:** 2022-06-06

**Authors:** Qiang Yue, Chao Wei

**Affiliations:** China National Basketball Academy, Shandong Sport University, Rizhao, Shandong 250102, China

## Abstract

With the development of information management of sports events, basketball teams have higher requirements for the management of training data. The development of data visualization technology has provided convenience for information management which evolves from a digital management model to an efficient graphical management model. In this article, we will mainly design a human body positioning system based on wireless sensor networks. The weak signal from the sensor is tuned by the circuit, and the cylindrical Fresnel lens array is selected to modulate the field of view to ensure an effective response to the infrared signal of the moving human body. The wireless sensor network is used to integrate the human body detected by each pyroelectric sensor node, and the infrared signal is sent to the upper computer for analysis and processing. Through the host computer interface, observe and analyze the relationship between the detection signal of a single pyroelectric sensor and the position, speed, and movement of the moving human body, and deeply explore the operating process of the wireless pyroelectric infrared sensor network system. With the development of the new era, Chinese basketball training and teaching methods generally have some drawbacks, which have seriously affected the quality and effect of college basketball training and restricted the development of college basketball. Therefore, the focus of research is to solve these problems in order to achieve more effective college basketball training and education effects. This article mainly through the research of 5G technology wireless sensing human body positioning shows that wireless sensing technology has made great progress in its aspects, systematically analyzes basketball training, and proposes better training methods.

## 1. Introduction

The progress and development of science and technology are driving people's demand for smart home life to gradually increase. In recent years, due to the development of Internet of Things technology, the field of smart home has developed rapidly [1]. 5G technology is designed to connect various Internet of Things devices to the network to achieve information integration, information monitoring, and information management functions, which makes the information exchange between all interconnected nodes more convenient, and human detection and positioning technology are used as a smart home, and the important part of the world is becoming more and more important [2]. At present, the methods used for human body positioning and recognition mainly include image-based human target recognition and high-frequency-based positioning and recognition technology [3]. Image-based detection and positioning algorithms are too complex, and the equipment is expensive and difficult to cope with the complex and changeable external environment. Radio frequency-based positioning technology is not yet fully mature and is susceptible to other radio frequency interference. Pyroelectric infrared sensors (PIR) are inexpensive, low power consumption, easy to install and deploy, can effectively detect human infrared signals, and can be combined with wireless sensor networks and machine learning technology, that is, indoors [4]. The research and application of human perception of it are becoming more and more extensive [5]. Pyroelectric infrared sensors are currently mainly used in the fields of smart lighting and security monitoring due to their low price and relatively mature technology. But the original output of the pyroelectric sensor is related to the difference in heat source, movement position, movement speed, and so on. Therefore, it is necessary to establish a robust and effective human body heat source identification and positioning system [6]. Since the human body itself is a heat source signal, it can emit thermal infrared signals (8∼14 *μ*m). Therefore, the construction of a pyroelectric infrared sensor network can effectively detect the energy in the designated field of view [7]. The heat source is moved. By analyzing the infrared signal of the induction, the motion state of the human body (position, direction, speed, posture) can be judged. Therefore, the design of a mobile human monitoring system based on a pyroelectric sensor network is very important for the positioning and detection of human motion in indoor space [8]. Therefore, this paper designs a human body infrared signal detection node, establishes a pyroelectric sensor network system model, and proposes a signal feature extraction method to overcome the shortcomings of a high error rate of target human body position detection [9]. Finally, this article reviews in detail the strategies for improving basketball training and the education of college students in the new era, examines the development and current situation of college basketball training in the new era, and examines common methods and misunderstandings in educational activities [10]. Next, we will effectively promote the development and progress of college basketball courses from three aspects: multilevel teaching, collaborative training, and fun training.

## 2. Related Work

The literature introduces the possibility of pyroelectric sensors of different heights in the detection and classification of human motion [11]. According to the different infrared distribution characteristics of different moving human bodies, fast Fourier transform, short-time Fourier transform, wavelet transform, and other algorithms are used [12]. The infrared signal characteristics of the human body are extracted. By fusing the different signal characteristics of multiple sensors with the CCA multifunctional fusion analysis algorithm, the classification and recognition accuracy of the human body within the detection range of 6 meters reaches 88.75%. The literature introduces the problems of pyroelectric infrared sensors in the application of human body positioning and proposes a method of realizing positioning by scanning the space with a rotating sensor to solve the problem of not being able to find a stationary human body. By using the shielding cover to limit the viewing angle of the sensor, the resolution of a single sensor in the system is improved, and the system can realize high-precision positioning of the human body [13]. The literature introduces a pyroelectric infrared sensor array placed on the ceiling. The SukLee design system has a positioning accuracy of up to 0.5 m, excellent real-time performance, and locates moving human bodies. SukLee solves the problem of stationary human body recognition by analyzing the human body movement pattern throughout the process, aiming at the problem of not being able to recognize the stationary human body, to a certain extent, but this method of analyzing the context data process is that the analysis method is cumbersome, and the real-time performance is not high [14]. A human body positioning system based on pyroelectric infrared sensors is being developed. According to the output characteristics of the sensor, design the signal processing circuit and data acquisition device, design the infrared signal acquisition node, quantify the electrical signal output of the sensor, send it to the gateway through the radio module, and finally upload it to the PC for processing. Finally, by analyzing the characteristics of the output signal of the sensor, designing a signal extraction algorithm, extracting the human body infrared signal from the noise of the environment, designing a human body positioning algorithm, and obtaining the human body position coordinates by analyzing and processing the human body infrared signal.

## 3. Research on Human Body Positioning System Based on 5G Wireless Sensing Technology

### 3.1. The Overall Design of the Human Body Positioning Recognition System

**Figure 1:**
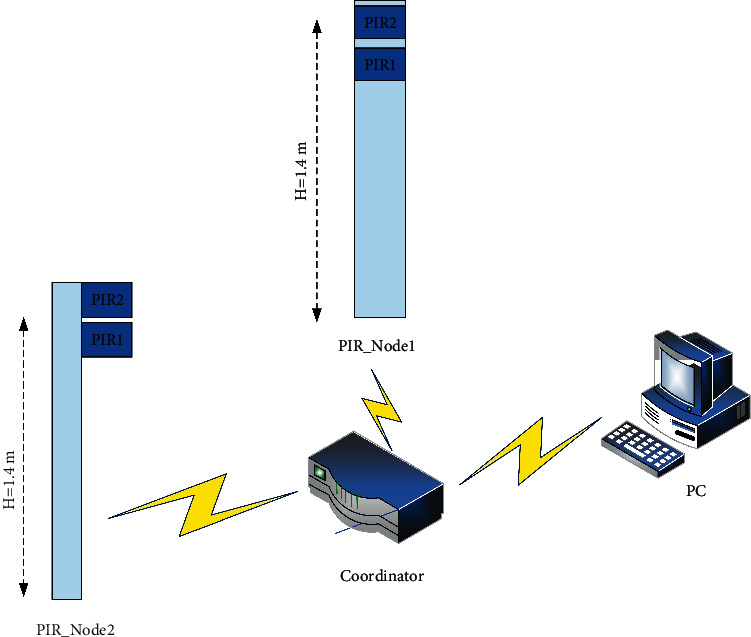
Overall block diagram of the system.

Figure 1 shows the overall design block diagram of the system. Each PIR sensor end node contains two PIR sensors, composed of a pyroelectric probe and a cylindrical Fresnel lens array. The Fresnel lens array can provide the functions of the field of view modulation (FOV) and heat source focusing. The pyroelectric probe is a direct measurement of human thermal radiation. A wireless sensor network is formed between the two PIR sensor end nodes and the coordinator node to make the network, and the data transmission and reception of each node in it become possible. The coordinator node receives the collected wireless pyroelectric sensor data frame, processes the data, and sends it to the PC. The PC completes the package and storage of the received data frame time sequence through the Matlab program and performs the packaged signal time sequence data preprocessing, neural network training, and signal prediction classification. In addition, the waveform signals generated during human movement can be monitored in real time through the PC interface, and these signals can be analyzed to extract useful signal characteristics.

**Figure 2:**
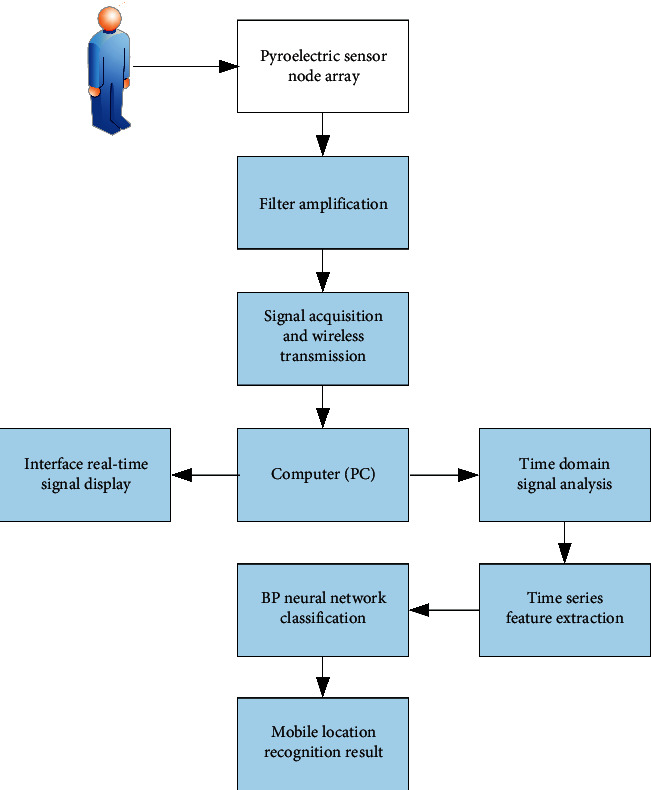
Flow chart of mobile human position recognition.

This subject uses the principle that the pyroelectric infrared sensor can detect the infrared signal of the moving human body, with the purpose of detecting the location of indoor immigrants, and a human body positioning system based on the wireless pyroelectric infrared sensor network is developed.  Figure 2 shows the position detection process of the movable human body.

An artificial neural network (ANN) is a theoretical mathematical model that simulates the mechanism of the human brain. A simple neuron model is created through the abstraction of human brain neurons. The neurons are connected to each other through a neural network, and the network is formed according to an appropriate connection method. Constructing a large-scale nonlinear adaptive system from input to output can amplify or suppress the transmitted signal by adjusting the weight and threshold of the connections between neurons. At this stage, neural networks have been successfully applied to many research fields such as signal processing and decision support.

As the basic unit of the neural network, the neuron is responsible for information processing and transmission and is generally abstracted as a nonlinear device with multiple inputs and a single output.

The mathematical model of any neuron can be expressed as

(1) $$\begin{array}{c} y=f\left(\sum_{i=1}^{n}w_{ij}x_{i}-\theta \right). \end{array}$$

In a basic neuron model, commonly used activation functions include

(2) $$\begin{array}{c} \mathrm{sigmoid}\left(x\right)=\frac{1}{1+e^{-\partial x}}. \end{array}$$

The hyperbolic tangent function is as follows:

(3) $$\begin{array}{c} \tanh\left(x\right)=\frac{e^{\partial x}-e^{-2x}}{e^{\partial x}+e^{-\partial x}}. \end{array}$$

The forward propagation process of the neural network is as follows:

(4) $$\begin{array}{c} \partial_{1}^{\left(2\right)}=f\left(W_{11}^{\left(1\right)}x_{1}+W_{12}^{\left(1\right)}x_{2}+W_{13}^{\left(1\right)}x_{3}+b_{1}^{\left(1\right)}\right), \end{array}$$

(5) $$\begin{array}{c} \partial_{2}^{\left(2\right)}=f\left(W_{21}^{\left(1\right)}x_{1}+W_{22}^{\left(1\right)}x_{2}+W_{23}^{\left(1\right)}x_{3}+b_{2}^{\left(1\right)}\right), \end{array}$$

(6) $$\begin{array}{c} \partial_{3}^{\left(2\right)}=f\left(W_{31}^{\left(1\right)}x_{1}+W_{32}^{\left(1\right)}x_{2}+W_{33}^{\left(1\right)}x_{3}+b_{3}^{\left(1\right)}\right), \end{array}$$

(7) $$\begin{array}{c} h_{W,b}\left(x\right)=\partial_{1}^{\left(3\right)} \end{array}$$

It can be seen from the formula (4)–(7) that in the forward propagation, the previous neuron is used as the input of the secondary neuron, and the output of the network can be changed by changing the connection weight and bias term.

The mean square error used in the neural network is

(8) $$\begin{array}{c} J\left(W,b;x^{\left(i\right)},y^{\left(i\right)}\right)=\frac{1}{2}{\left\|h_{w,b}\left(x^{\left(i\right)}\right)-y\left(i\right)\right\|}^{2}. \end{array}$$

If the training set contains *N* training samples, the total loss function of the neural network is

(9) $$\begin{array}{c} J\left(W,b\right)=\left[\frac{1}{N}\sum_{i=1}^{N}J\left(W,b;x^{\left(i\right)},y^{\left(i\right)}\right)\right]+\frac{\lambda^{n_{1}-1}}{2}\sum_{l=1}^{s_{l}}\sum_{i=1}^{s_{i}+1}\sum_{j=1}^{N}{\left(W_{jl}^{\left(l\right)}\right)}^{2}\\ =\left[\frac{1}{N}\sum_{i=1}^{N}\left(\frac{1}{2}{\left\|h_{w,b}\left(x^{\left(i\right)}\right)-y\left(i\right)\right\|}^{2}\right)\right]+\frac{\lambda^{n_{n}-1}}{2}\sum_{l=1}^{s_{l}}\sum_{i=1}^{s_{l}}\sum_{j=1}^{s_{l}+1}{\left(W_{ji}^{\left(j\right)}\right)}^{2}. \end{array}$$

The process of using the gradient descent algorithm to update the network weight parameter *W* and the bias parameter *b* in the BP neural network is as follows:

(10) $$\begin{array}{c} W_{ij}^{\left(l\right)}=W_{ij}^{\left(l\right)}-\beta \frac{\partial }{\partial W_{ij}^{\left(l\right)}}J\left(W,b\right), \end{array}$$

(11) $$\begin{array}{c} b_{i}^{\left(l\right)}=b_{i}^{\left(l\right)}-\beta \frac{\partial }{\partial b_{i}^{\left(l\right)}}J\left(W,b\right). \end{array}$$

The partial derivatives of the network parameters in (9) and (10) are

(12) $$\begin{array}{c} \frac{\partial }{\partial W_{ij}^{\left(l\right)}}J\left(W,b\right)=\left[\frac{1}{N}\sum_{i=1}^{N}\frac{\partial }{\partial W_{ij}^{\left(l\right)}}J\left(W,b;x^{\left(i\right)},y^{\left(i\right)}\right)\right]+\lambda W_{ij}^{\left(l\right)},\\ \frac{\partial }{\partial b_{i}^{\left(l\right)}}J\left(W,b\right)=\frac{1}{N}\sum_{i=1}^{N}\frac{\partial }{\partial b_{i}^{\left(l\right)}}J\left(W,b;x^{\left(i\right)},y^{\left(i\right)}\right). \end{array}$$

The remaining part of each output neuron *i* in the *ni* layer of the neural network can be calculated as follows:

(13) $$\begin{array}{c} \delta_{i}^{\left(n_{l}\right)}=\frac{\partial }{\left.\partial_{{\bar{i}}_{i}}^{\left(n_{l}\right.}\right)}\frac{1}{2}{\left\|y-h_{W,b}\left(x\right)\right\|}^{2}\\ =-\left(y_{i}-a_{i}^{\left(n_{l}\right)}\right)\cdot f^{\prime }\left(z_{i}^{\left(n_{l}\right)}\right). \end{array}$$

Among them, the formula for calculating the remainder of the *i*-th neuron in the *i-*th layer is as follows:

(14) $$\begin{array}{c} \delta_{i}^{\left(l\right)}=\left(\sum_{j=1}^{s_{l}+1}W_{ji}^{\left(l\right)}\delta_{j}^{\left(l+1\right)}\right)\cdot f^{\prime }\left(z_{i}^{\left(l\right)}\right). \end{array}$$

Finally, the partial derivatives of (15) and (16) can be calculated.

(15) $$\begin{array}{c} \frac{\partial }{\partial W_{ij}^{\left(l\right)}}J\left(W,b;x,y\right)=a_{j}^{\left(l\right)}\delta_{i}^{\left(l+1\right)}, \end{array}$$

(16) $$\begin{array}{c} \frac{\partial }{\partial b_{i}^{\left(l\right)}}J\left(W,b;x,y\right)=\delta_{i}^{\left(l+1\right)}. \end{array}$$

Input to the neural network:

(17) $$\begin{array}{c} \Phi =\left\{\left(x^{\left(1\right)},y^{\left(1\right)}\right),\left(x^{\left(2\right)},y^{\left(2\right)}\right),\ldots ,\left(x^{\left(N\right)},y^{\left(N\right)}\right)\right\}. \end{array}$$

Calculation:

(18) $$\begin{array}{c} \Delta W^{\left(l\right)}=\Delta W^{\left(l\right)}+\nabla_{W^{\left(t\right)}}J\left(W,b;x,y\right). \end{array}$$

Calculation:

(19) $$\begin{array}{c} \Delta b^{\left(l\right)}=\Delta b^{\left(l\right)}+\nabla_{b^{\left(i\right)}}J\left(W,b;x,y\right). \end{array}$$

Update network parameters:

(20) $$\begin{array}{c} W^{\left(l\right)}=W^{\left(l\right)}-\alpha \left[\left(\frac{1}{m}\Delta W^{\left(l\right)}\right)+\lambda W^{\left(l\right)}\right],\\ b^{\left(l\right)}=b^{\left(l\right)}-\alpha \left(\frac{1}{m}\Delta b^{\left(l\right)}\right). \end{array}$$

### 3.2. Human Body Positioning and Recognition Algorithm

Support vector machine algorithm (SVM) is a statistical learning method that solves discrimination and regression problems by determining the hyperplane with the largest margin in the training sample. Improved methods on this basis include soft interval SVM and regression support vector regression (SVR) model.

For regression problems, for the training sample set,

(21) $$\begin{array}{c} D=\left\{\left(x_{1},y_{1}\right),\left(x_{2},y_{2}\right),\ldots ,\left(x_{n},y_{n}\right)\right\}. \end{array}$$

The SVR regression problem can be transformed into a problem of determining the minimum value of the objective function.

(22) $$\begin{array}{c} R\left(w,b\right)=\min\frac{1}{2}{\left\|w\right\|}^{2}+C\sum_{i=1}^{m}l_{t}\left(f\left(x_{i}\right)-y_{i}\right). \end{array}$$

The loss function with the introduction of relaxation factors is as follows, and equation (22) can be rewritten as

(23) $$\begin{array}{c} l_{t}\left(z\right)=\left\{\begin{array}{cc} 0, & \text{if }\left|z\right|\leq \varepsilon ,\\ \left|z\right|, & \text{otherwise,} \end{array}\right.\\ R\left(w,b,\xi_{i},{\hat{\xi }}_{i}\right)=\min\frac{1}{2}{\left\|w\right\|}^{2}+C\sum_{i=1}^{m}\left(\xi_{i}+{\hat{\xi }}_{i}\right). \end{array}$$

The constraints are

(24) $$\begin{array}{c} \text{s.t. }\left\{\begin{array}{c} f\left(x_{i}\right)-y_{i}\leq \varepsilon +\xi_{i},\\ f\left(x_{i}\right)-y_{i}\leq \varepsilon +{\hat{\xi }}_{i},i=1,2,\ldots ,m,\\ \xi_{i}\geq 0,{\hat{\xi }}_{i}\geq 0. \end{array}\right. \end{array}$$

The introduction of Lagrangian multipliers provides it with the following Lagrangian functions:

(25) $$\begin{array}{c} L\left(w,b,\alpha ,\hat{\alpha },\xi ,\hat{\xi },\mu ,\hat{\mu }\right)={\left\|w\right\|}^{2}+C\overset{m}{\underset{i=1}{\sum }}\left(\xi_{i}+{\hat{\xi }}_{i}\right)-\overset{m}{\underset{i=1}{\sum }}\mu_{i}\xi_{i}-\overset{m}{\underset{i=1}{\sum }}{\hat{\mu }}_{i}{\hat{\xi }}_{i}\\ C\overset{m}{\underset{i=1}{\sum }}\left(\xi_{i}+{\hat{\xi }}_{i}\right)-\overset{m}{\underset{i=1}{\sum }}\mu_{i}\xi_{i}-\overset{m}{\underset{i=1}{\sum }}{\hat{\mu }}_{i}{\hat{\xi }}_{i}\\ \overset{m}{\underset{i=1}{\sum }}\alpha_{i}\left(f\left(x_{i}\right)-y_{i}-\varepsilon -\xi_{i}\right)+\overset{m}{\underset{i=1}{\sum }}\alpha_{i}\left(y_{i}-f\left(x_{i}\right)-\varepsilon -{\hat{\xi }}_{i}\right). \end{array}$$

Finally, the result is introduced into the Lagrangian function to solve the SVR dual problem, and we get

(26) $$\begin{array}{c} W\left(\alpha_{i},{\hat{\alpha }}_{i}\right)=\max\sum_{i=1}^{m}y_{i}\left({\hat{\alpha }}_{i}-\alpha_{i}\right)-\varepsilon \left({\hat{\alpha }}_{i}+\alpha_{i}\right)-\frac{1}{2}\sum_{i=1}^{m}\sum_{j=1}^{m}\left({\hat{\alpha }}_{i}-\alpha_{i}\right)\left({\hat{\alpha }}_{j}+\alpha_{j}\right)x_{i}^{T}x_{j}. \end{array}$$

The sensor node is mainly composed of an MCU data acquisition unit, several PIR sensor units, and a wireless data transmission unit. A sensor node model is designed in the system to measure the movement position and velocity characteristics of the human target. The sensor node is composed of two orthogonal infrared sensor units in the field of view, which is used to set the field of view orthogonal to the horizontal and vertical directions and makes it possible to more accurately detect the state, direction, and position of human movement. The cylindrical Fresnel lens array is selected to define the field direction and area. In order to effectively detect the infrared signal of the human body, the number, position, and external field of view of the sensor node need to be set, which is directly related to the field of view and detection of the sensor node effect.

In the sensor node design, each sensor node contains N PIR sensors, and the relationship between the voltage output *v*_*i*_ of the *i*-th PIR sensor and the field of view of the sensor is as follows:

(27) $$\begin{array}{c} v_{i}=f\left(\overset{M}{\underset{j=1}{\sum }}\phi_{i,j}\cdot x_{j}\right). \end{array}$$

*ϕ*_*i,j*_ is expressed as follows:

(28) $$\begin{array}{c} \phi_{i,j}=\left\{\begin{array}{c} 1,\text{visible, }\\ 0,\text{invisible. } \end{array}\right. \end{array}$$

### 3.3. Simulation Experiment Results and Analysis

For this circuit, the PIR probe chooses the D203S model, and its specifications are listed in Table 1.

**Figure 3:**
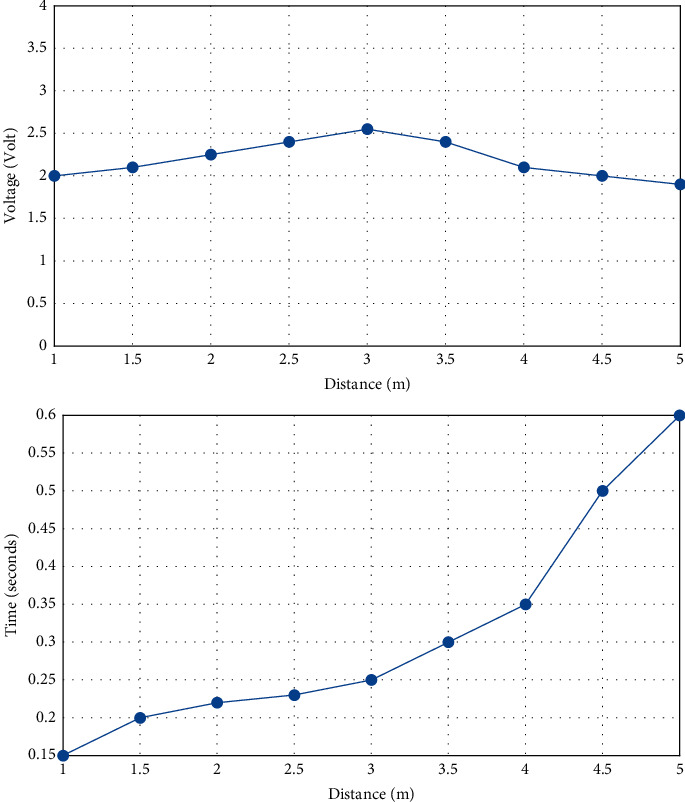
The relationship between the sensor output signal and horizontal movement distance.

Experiments show that when the human body moves at the same speed according to the output of PIR1, different distances will affect the output of the sensor. In this experiment, the amplitude of the sensor output signal is the largest at the distance *D* = 3 m, and the average peak time difference also increases with the increase of the distance.  Figure 3 shows the relationship between the sensor output signal peak value and the peak time difference and the horizontal travel distance.

**Figure 4:**
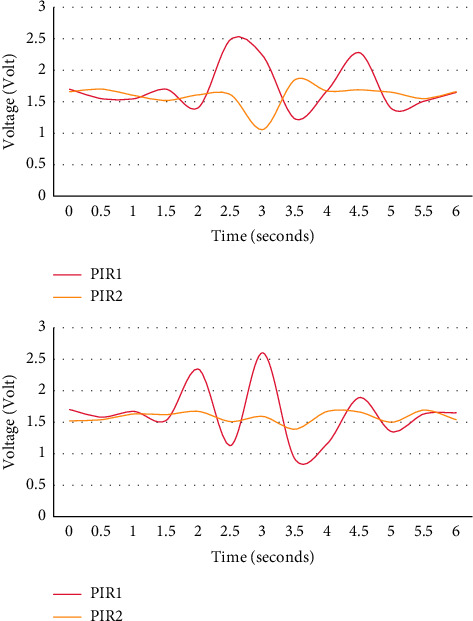
Sensor output waveforms at different speeds and the same distance.

As shown in Figure 4, the left side is the real-time signal waveform captured by the moving path R2 at a normal speed, and the right side is the real-time signal waveform captured by the moving path R2.

It can be seen from the output signal of PIR1 in the experiment that when the human body follows the same path, different movement speeds will affect the output signal of the sensor. In this experiment, the higher the speed, the smaller the average amplitude of the sensor output, and the higher the speed, the smaller the average peak time difference.

**Figure 5:**
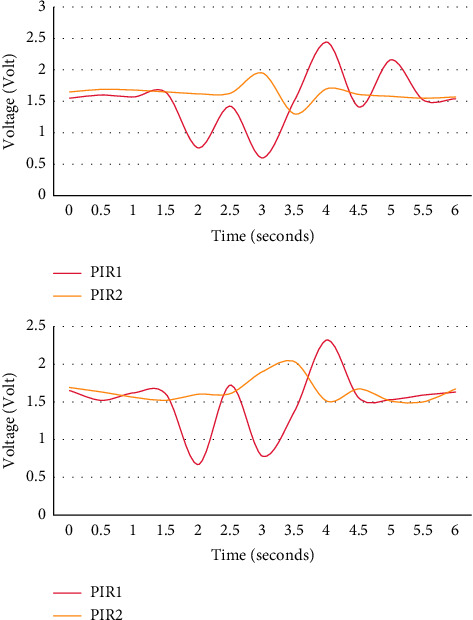
Sensor output waveforms at the same speed at different moving angles.

As shown in Figure 5, the left side is the real-time signal waveform captured by the normal-speed moving path R5, and the right side is the real-time signal waveform captured by the normal-speed moving path R6.

Experiments show that at the same speed of the human body, different motion angles will affect the output signal of the sensor according to the output signal of PIR1. In the experiment, the tracks of R5 and R6 overlap, but the direction of motion is opposite. As the human body moving on the path R5 gradually moves away from the sensor node at a specific inclination angle, the peak time series shows a specific trend. The first peak direction of the sensor output signal in the path R6 is opposite to the path R5, and the peak time series is also there are many opposite trends.

**Figure 6:**
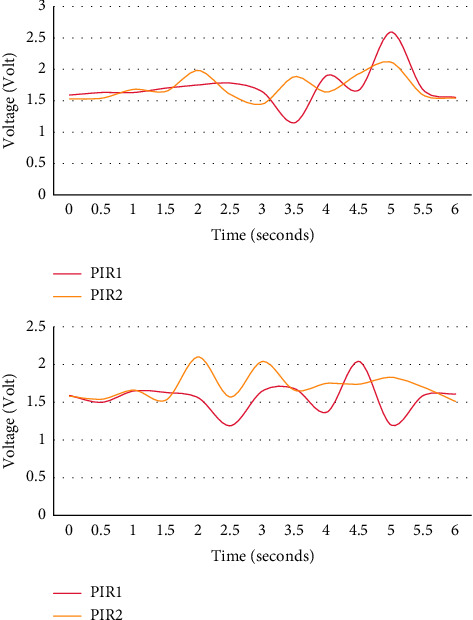
Sensor output waveforms under different actions at the same position.

As shown in Figure 6, the left side is the real-time signal waveform captured by the two crouching motions facing the S position sensor node, and the right side is the real-time signal waveform sensor captured by the stepping motion facing the sensor node at S.

Experiments show that when the human body performs different actions at the same position, PIR1 and PIR2 will produce different output waveforms due to different actions, and the characteristics of the waveform sequence can reflect the action information well.

As shown in Table 2, the recognition results of the original data set and the peak time series feature data set (simplified features) are analyzed, and the recognition results of single knots and double knots are analyzed. The original data dimension is 600, and the simplified feature dimension is 16. It can be seen from the experimental results that the detection rates of node 1 and node 2 are similar. This is because each node has a high detection rate when detecting horizontal movement and a high detection rate when detecting vertical movement. When two nodes work at the same time, the overall visibility of the network is high due to the data fusion between the nodes. If the original data set is used, the time series retrieved in the same path will be very different, because the starting point of the time series is blocked at different positions, but the peak time series function does not have these problems and realizes the detection of the position of the moving human body. It has a 95.30% correct rate.

## 4. Innovative Research on Basketball Training System and Teaching Strategy under the Internet Environment

### 4.1. Design of Basketball Training Network Auxiliary System

**Figure 7:**
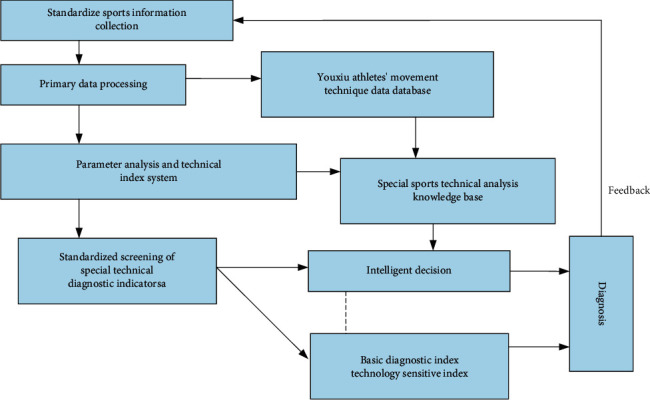
The working mode of the diagnostic system for elite athletes.

In today's complex and modern competitive sports, computer technology has attracted more and more attention from sports professionals. The “scientific” training of athletes has been verified and further developed in various sports events (Olympics, World Championships, National Games). Develop and build a complete set of the systematic and complete training system, as shown in Figure 7. However, the current technical requirements for virtual reality software and hardware are more limited. Various special interactive devices based on virtual reality are very expensive. The existing interactive methods are not humane enough, the operation is more complicated, and the real-time system performance and accuracy are not high. These restrictions are serious. It limits the widespread use and application of virtual reality technology in the field of real sports training. With the continuous progress of virtual reality software and hardware technology, we can expect more innovations and more applications in the near future.

The design of the database entity table is based on the principle of generality, so that it can be applied to other sports in the future. The training data are stored in the database table. The database has four tables: date table, score table, index table, and athlete table. The structure is shown in the following table.

This table is used to store ID and some data.  Table 3 describes the attribute fields in the date table.

This table is mainly used to store the scores of different types of indicators, such as ID, score, date ID, athlete ID, and indicator ID.  Table 4 describes the attribute fields in the score table.

This table is mainly used to store information such as the ID, index name, and index type of the training index.  Table 5 describes the attribute fields in the indicator table.

This table is mainly used to store basic athlete information, such as ID, name, age, gender, phone number, and class.  Table 6 describes the attribute fields in the athlete table.

### 4.2. Exploration of Basketball Training and Teaching Methods in the New Era

College students' basketball training and education activities are different from other professional sports, and they put high demands on the personal physique and physical coordination training of college students. Therefore, basketball training and education activities usually focus on basketball skills, physical fitness training, and physical fitness adjustment skills. First, the intensity of basketball training and development of basketball skills are higher than the depth of education content when most professional teachers set up this educational content. The focus is on dribbling, layup, shooting, and other skills training for college students. The teaching in this area is mainly based on the training of students' physical coordination ability. Through the planning and implementation of running training such as dribbling and passing, it can improve students' sense of the ball, improve the coordination of all aspects of training, improve physical fitness, and improve training level. Second, in the context of the new era, the physical training methods used in basketball training and educational activities for college students are the same as the content of basketball skills education. Generally speaking, physical training for college students is based on the requirements of basketball skills training and focuses on college students' physical exercise. Courses at this stage usually focus on college basketball explosiveness and endurance training, especially upper and lower limb training for college students.

Basketball is a team sport, and it is very important in basketball games, because basketball is mainly based on teamwork. No matter how good a basketball player's personal skills are, he cannot win the game or succeed without the help of his teammates. As a basketball sport with team fighting attributes, good team tactics and tactical cooperation between teams are the basic requirements to ensure that the basketball team wins the game. Therefore, when choosing college basketball training and teaching methods, most professional basketball teachers not only pay attention to college basketball skills and physical training but also focus on overall team combat training. The training activities, physical fitness training, and teaching content of the college basketball team's comprehensive combat ability also include single-game tactics and strategic match coordination training. Tactical coordination training is mainly carried out through long-term simulation game scenes in cooperation with the university basketball team. Based on the implicit understanding between teammates, there is no need to adjust the game style, and the rhythm of the game can also be adjusted. The single-game training strategy pays more attention to dynamic information such as running positions, pick-and-rolls, empty positions, and scoring. This is a strategic deployment to control the rhythm of the game. College students need long-term training and courses to strengthen the collaboration between team members.

### 4.3. Research on Basketball Training and Teaching Innovation Strategies in the New Era

In order to implement the new educational concept of “student-oriented, teaching students in accordance with their aptitude,” the pertinence of college basketball education and education in the new era and teaching methods and basic skills should be improved. First, regularly evaluate the physical fitness, physical fitness, physical coordination ability, and basketball skills of college students, and based on the survey and monitoring results, formulate basketball training methods and courses that meet the physical condition and development needs, and introduce various types of basketball training that suit each student's personality. Basketball training and teaching make educational activities more relevant.

Secondly, good basic basketball skills promote the steady improvement of college students' basketball skills and achieve more efficient basketball training and education effects. Therefore, professional basketball teachers in the new era should aim at improving the effect of college students' basic basketball training and organize exciting, responsive, and intelligent basketball education activities. Basketball training and teaching should be divided into several stages: “implementation of stepped teaching” and “step-by-step teaching”. At the end of each stage of basketball training and educational activities, students should be evaluated based on the actual situation of completing basketball training. Through this opportunity, teachers can understand their own basketball skills, make corrections and improvements in time, and help students consolidate their basic skills.

Professional basketball coaches aim to improve the tactical strategy and team coordination of college students in the new era and aim to improve the coordination between college students and basketball team members. They must divide their teams wisely. Coordination and foundation among team members through collaborative group training, it is ensured that college students and team members have a positive psychology to win basketball games and strengthen students' consensus on tactics. Secondly, we need to further strengthen our interpretation of on-court tactics in actual game scenes. Typical examples of NBA, CBA, Olympics, and other large-scale basketball events can be appropriately used as educational resources, multimedia educational equipment, information technology, and computer equipment. The courseware display format of pause, play, and slow motion at any time allows students to have enough time to understand the real game format and make subjective judgments. When college students make subjective decisions, professional basketball coaches guide students to adjust their tactics according to their own misunderstandings, improve their tactical coordination, and achieve more efficient basketball training.

## 5. Conclusion

The indoor infrared pyroelectric human body positioning detection system developed in this paper uses a wireless sensor network to capture and detect the original infrared signal of a single moving human body in the field of view and perform data processing and data fusion on it. By extracting the characteristic signal of the sensor in the time domain, the neural network classification algorithm is designed to realize the prediction and classification of the human body position. By studying the mechanism of pyroelectric infrared sensor and Fresnel lens array, an infrared sensor unit has been developed, which can effectively detect the infrared signal of the human body in the field of view. By analyzing the time-domain characteristics of the output signal of the infrared sensor unit, the relationship between the output signal of the pyroelectric infrared sensor and the position, speed, direction, and movement of the moving human body is studied. And this article also found some misunderstandings in the basketball training and teaching methods of Chinese college students in the context of the development of the new era, which will seriously affect the efficiency and quality of teaching, seriously affect the enthusiasm of college students to participate, and limit the enthusiasm of college students to participate in basketball training and educational activities. However, as long as professional basketball teachers recognize the root causes of these problems and take effective measures fundamentally, these common problems will be resolved to achieve the expected training and teaching effects.

## Figures and Tables

**Table 1:** D203S specifications.

Specification	Parameter
Window size	3 × 5 mm
Infrared receiving electrode	2 × 1 mm, 2elements
Encapsulation	TO-5
Accepted wavelength	5〜15 *μ*m
Transmittance	>76%
Sensitivity	>3500 V/W
Voltage	3–15 V
Operating temperature	−30∼+70°C
Angle of incidence	138° × l25°
Detection rate	1.4 × l0^8^ cmHz^1/2^W

**Table 2:** Classification and recognition results.

Data set category	Node l (%)	Node 2 (%)	Node l and node 2 (%)
Original data set	66.42	64.05	77.35
Simplified feature data set	81.26	82.82	95.30

**Table 3:** Date table.

Field name	Type of data	Primary key	Description
Id	Int	Yes	Date ID
mou_name	Varchar	No	Specific date

**Table 4:** Results table.

Field name	Type of data	Primary key	Description
Id	Int	Yes	Grade ID
rep_num	Varchar	No	Fraction
rep_mouth_id	Int	No	Date ID
rep student id	Int	No	Athlete ID
rep_subject_id	Int	No	Indicator ID

**Table 5:** Index table.

Field name	Type of data	Primary key	Description
Id	Int	Yes	Indicator ID
sub_name	Varchar	No	Indicator name
Sub_type	Varchar	No	Indicator type

**Table 6:** Athlete table.

Field name	Type of data	Primary key	Description
Id	Int	Yes	Athlete ID
stu_name	Varchar	No	Athlete's name
stu_age	Int	No	Athlete's age
stu_sex	Varchar	No	Athlete gender
stu_phone	Varchar	No	Athlete phone
stu_role	Varchar	No	Athlete level

## Data Availability

The data used to support the findings of this study are available from the corresponding author upon request.
